# Black Cumin Seed Oil Disrupts Structural Biomass and Attenuates Porphyrin-Associated Maturation in Mature Oral Microcosm Biofilms: An In Vitro Study

**DOI:** 10.3390/biomedicines14081874

**Published:** 2026-08-21

**Authors:** Ahyun Jo, Jiyeon Lee, A-Young Chun, Min-Kyung Kang, Hee-Eun Kim

**Affiliations:** 1 Department of Health Science, Gachon University Graduate School of Public Health, Incheon 21936, Republic of Korea; dkgus25864@gachon.ac.kr (A.J.); angel3327@gachon.ac.kr (J.L.); 2 Department of Health Science, Graduate School of Gachon University, Incheon 21936, Republic of Korea; dkdud2854@gachon.ac.kr; 3 Department of Dental Hygiene, Hanseo University, Seosan, Chungcheongnam-do 31963, Republic of Korea; kmk0709@hanseo.ac.kr; 4 Department of Dental Hygiene, Gachon University College of Medical Science, Incheon 21936, Republic of Korea

**Keywords:** anti-infective agents, chlorhexidine, dental biofilm, extracellular polymeric substances, *Nigella sativa*

## Abstract

**Background/Objective**: Mature oral biofilms resist conventional antiseptics via dense extracellular polymeric substance (EPS) matrices. Consequently, the prolonged use of broad-spectrum agents like chlorhexidine (CHX) raises profound ecological concerns. Using a mature oral microcosm biofilm model, we evaluated the multifaceted efficacy of 0.5% black cumin seed oil (BCSO) in disrupting structural biomass, suppressing aciduric bacterial viability, and attenuating porphyrin-associated anaerobic biofilm maturation. **Methods**: Saliva-derived microcosm biofilms were cultivated on hydroxyapatite discs in a mucin-containing medium (0.5% sucrose) for 6 days. Mature biofilms were treated daily for 1 min for 6 days (*n* = 19/group) with 0.5% BCSO, 0.12% CHX (positive control), or 0.5% dimethyl sulfoxide (negative control). EPS and bacterial biovolumes were quantified using confocal microscopy. Viability was strictly evaluated as aciduric bacterial colony-forming units. Pathogenic potential was specifically assessed as porphyrin-associated maturation by the red-to-green fluorescence intensity ratio (${\mathrm{Ratio}}_{R/G}$) using quantitative light-induced fluorescence-digital technology. **Results**: Compared with the negative control, 0.5% BCSO significantly degraded the EPS matrix (*p* < 0.001) and bacterial biovolumes (*p* = 0.045), and reduced aciduric bacterial counts (*p* = 0.028). Importantly, 0.5% BCSO significantly reduced the ${\mathrm{Ratio}}_{R/G}$ (*p* = 0.015), compared with the negative control, indicating severely attenuated porphyrin-associated anaerobic maturation. In the CHX group, the reduction did not reach statistical significance compared with the negative control (*p* = 0.399). **Conclusions**: In vitro, 0.5% BCSO and 0.12% CHX effectively reduced structural biomass and aciduric bacterial viability. Furthermore, BCSO significantly reduced late-stage, porphyrin-associated anaerobic maturation compared with the negative control, whereas the reduction observed with CHX did not reach statistical significance. These findings highlight the potential of BCSO as a targeted natural antibiofilm adjunct, warranting rigorous in vivo and mechanistic validation before clinical translation.

## 1. Introduction

Oral biofilms are highly organized, surface-attached microbial consortia embedded within a three-dimensional matrix of extracellular polymeric substances (EPS) synthesized primarily by oral microorganisms [1]. This matrix is not merely structural; it provides a scaffold that supports microbial adhesion, co-aggregation, and microcolony development [2,3] and acts as a diffusion barrier that restricts the penetration of antiseptics [4]. As biofilms mature, these properties give rise to two distinct treatment challenges: mechanical resistance owing to the increased viscoelasticity of the matrix [2,5] and chemical tolerance to the conventional antiseptics used as adjuncts [6].

In addition to these structural defenses, biofilm maturation is accompanied by complex biological shifts that further enhance its pathogenic potential [7]. While intercellular signaling networks and ecological disturbances drive biofilm maturation [8], the ultimate phenotypic barrier to clinical intervention is this structurally recalcitrant, dense EPS matrix [9]. By shielding a highly viable bacterial consortium, mature biofilms exhibit profound antimicrobial tolerance and persist in poorly accessible niches [10], thereby perpetuating chronic inflammation and tissue destruction [11]. Although mechanical debridement remains the first-line strategy for biofilm control, its effect is inherently incomplete because biofilm fragments and matrix-embedded communities persist at inaccessible sites [9]. This underscores the need for adjunctive antiseptic strategies that not only aid in physical removal but also effectively penetrate and disrupt the physical architecture of residual biofilms to mitigate their virulence and dysbiotic progression [2].

For this purpose, chlorhexidine (CHX) remains the most extensively utilized adjunctive antiseptic, serving as the gold standard for chemical plaque control in oral environments. However, because its antibacterial action is nonspecific and broad-spectrum, prolonged use may disturb the commensal oral microbiota and promote dysbiosis [12]. This concern has been further amplified by the widespread application of CHX as a pre-procedural rinse during the COVID-19 pandemic, which coincided with reports of reduced CHX susceptibility among oral bacteria [13]. Coupled with the increasing detection of resistant taxa within oral biofilms [14], there is a compelling need for novel antibiofilm adjuncts. Ideal alternatives should effectively dismantle mature biofilms while exerting substantially lower ecologically selective pressure on the commensal microbiome than conventional broad-spectrum antiseptics.

In this context, natural product-based antibacterial adjuncts have attracted attention as potential therapeutic candidates with a lower propensity to drive resistance [15], and a wide range of plant-derived compounds has been investigated for their antibiofilm effects [16]. Among these, black cumin seed oil (BCSO), obtained from the seeds of *Nigella sativa*, has a long history of medicinal use in Middle Eastern and Asian countries [17]. Its principal bioactive constituent, thymoquinone, exhibits antibiofilm activity against various dental pathogens, including multidrug-resistant strains, through mechanisms such as reactive oxygen species generation and bacterial membrane disruption [18]. It also possesses anti-inflammatory and antioxidant properties [19,20]. Importantly, while isolated thymoquinone drives much of this activity by compromising matrix integrity and EPS production [21], utilizing the whole extract (BCSO) offers distinct translational advantages driven by potential complementary or multi-component effects [22]. Whole botanical oils often exert such multi-target actions due to the presence of complementary secondary metabolites [23,24,25], potentially enhancing matrix penetration compared to single-molecule applications. Furthermore, as a natural oil, BCSO presents favorable in vitro cytocompatibility, supporting its potential for eventual incorporation into oral care formulations [19,26]. This aligns with the broader reorientation of biofilm control, from mere bacterial killing to the structural disruption of the supporting matrix and reduction in aggregate viability [27]. Collectively, these properties support the potential of BCSO as a promising natural adjunct for controlling mature oral biofilms.

While previous studies have reported the antibiofilm activity of BCSO against oral pathogens such as *Streptococcus mutans* [17,18], these investigations have primarily relied on single-species models [21,28] or early stage biofilms [26]. Such models do not capture interspecies interactions [29] or the robust structural stability, dense EPS matrix, and increased antimicrobial tolerance observed in vivo [7,24]. Consequently, it remains unclear whether the antibiofilm and matrix-disrupting effects of BCSO are preserved in clinically relevant mature oral biofilms. To address this limitation, we used a human saliva-derived oral microcosm model that preserves the structural heterogeneity and ecological complexity of the oral cavity [30,31]. Therefore, using a mature oral microcosm model, we aimed to evaluate the multifaceted efficacy of 0.5% BCSO in disrupting structural biomass, selectively suppressing the viability of aciduric populations, and ultimately attenuating the pathogenic potential of the biofilm.

## 2. Materials and Methods

### 2.1. Ethics Statement and Informed Consent

Ethical approval for this study was obtained from the Institutional Review Board of Gachon University (approval no. 1044396-202312-HR-237-01), and all procedures were performed in accordance with the principles of the Declaration of Helsinki. Before saliva was collected, the participants received an explanation of the study objectives and procedures and subsequently provided written informed consent.

### 2.2. Sample Size Calculation and Group Allocation

Sample size estimation was performed based on pilot data using G*Power v3.1.9.7 (Heinrich Heine University, Düsseldorf, Germany). Assuming a statistical power of 80%, an effect size (f) of 0.43, and a significance level (α) of 0.05 for one-way analysis of variance (ANOVA), the required total sample size was calculated as 57. Accordingly, 19 samples were assigned to each of the three groups (negative, experimental, and positive control).

### 2.3. Saliva Donor Selection and Processing

To strictly standardize the baseline microbiological architecture and deliberately eliminate the complex confounding effects of interindividual microbiome variability during the initial observation of treatment efficacy, stimulated whole saliva was collected from a single systemically and orally healthy female donor (aged 24 years). The inclusion criteria for the donor required the complete absence of active cavitated carious lesions, a gingival index of 0, and no history of systemic antibiotic therapy within the preceding three months. To further minimize variability in the inoculum, the donor avoided tooth brushing and other oral hygiene procedures for 24 h before sampling. Salivary flow was induced by chewing unflavored, sugar-free paraffin wax, and saliva was collected and passed through sterile glass wool (Kanto Chemical, Tokyo, Japan) to remove debris.

### 2.4. Chemical Characterization and Biological Optimization of BCSO

#### 2.4.1. Quantification of Thymoquinone by High-Performance Liquid Chromatography Analysis

The absolute thymoquinone content of cold-pressed BCSO (PREMIUM Black Seed Oil, Amazing Herbs, Buford, GA, USA; Batch/Lot No. CA0424231) was determined using a ChroZen high-performance liquid chromatography (HPLC) system (YoungIn Chromass Co., Ltd., Anyang, Republic of Korea). Prior to analysis, the oil sample was diluted in acetonitrile, vortex-mixed thoroughly, and filtered through a 0.45 μm membrane filter. All sample preparation procedures were performed under light-protected conditions to minimize the photodegradation of thymoquinone [32]. Chromatographic separation was performed on a Capcell Pak C18 MG II column (4.6 × 250 mm, 5 μm; Osaka Soda Co., Ltd., Osaka, Japan) maintained at 30 °C. The mobile phase consisted of acetonitrile and distilled water (82:18, *v*/*v*) under isocratic elution at a flow rate of 0.9 mL/min [32]. A 10 μL aliquot of each sample was injected, and thymoquinone was detected at 254 nm using a UV-visible absorbance detector. Chromatographic data were acquired and processed using the AutoChro-3000 software (YoungIn Chromass Co., Ltd., Anyang, Republic of Korea). The identification of thymoquinone was achieved by comparing the retention time of the sample peak with that of an authentic thymoquinone reference standard (≥98% purity, Sigma-Aldrich, St. Louis, MO, USA). Absolute quantification was performed using an external calibration curve generated from serial dilutions of thymoquinone standard. The thymoquinone concentration was expressed as µg/mL of BCSO. All HPLC analyses were performed in triplicates.

#### 2.4.2. Optimization of BCSO Concentration: Antimicrobial and Cytotoxic Properties

The optimal BCSO concentration was determined using disk diffusion assay. A stock solution of cold-pressed BCSO was diluted in 0.5% dimethyl sulfoxide (DMSO) to achieve final concentrations of 0.25, 0.5, and 1%. To ensure uniform dispersion, the samples were sonicated for 5 min (SHB-1025; Saehan Sonic, Seoul, Republic of Korea) and vortex-mixed (VM-96A; Lab Companion, Seoul, Republic of Korea). Sterile paper discs (6 mm diameter) were impregnated with 10 μL of each BCSO solution (0.25%, 0.5%, and 1%) and air-dried. The filtered saliva was inoculated onto brain heart infusion agar plates, and the discs were placed equidistantly on the agar surface. The plates were incubated at 37 °C in 10% CO_2_ for 48 h, after which the inhibition zone diameters were measured using a digital caliper.

To evaluate eukaryotic cytocompatibility, cytotoxicity was assessed using the MTT assay in accordance with ISO 10993-5 standards [33]. L929 murine fibroblast cells (ATCC CCL-1; American Type Culture Collection, Manassas, VA, USA) were dispensed at a density of 1 × 10^4^ cells/well into 96-well plates containing RPMI 1640 medium (Gibco Laboratories, Grand Island, NY, USA) supplemented with 10% fetal bovine serum and 1% penicillin-streptomycin. The cells were incubated for 24 h before exposure to different BCSO concentrations and a vehicle control containing 0.5% DMSO. After 24 h, the cells were washed with phosphate-buffered saline (PBS; Welgene, Gyeongsan, Republic of Korea) and treated with 2,5-diphenyl-2H-tetrazolium bromide (MTT) solution (1 mg/mL) for 2 h in the dark. The resulting formazan crystals were solubilized with isopropanol (Sigma-Aldrich, St. Louis, MO, USA), and the absorbance at 570 nm was measured using a spectrophotometer (CM-3500d; Minolta, Tokyo, Japan). Concentrations at which cell viability exceeded 70% were considered noncytotoxic [33].

### 2.5. Oral Microcosm Biofilm Formation and Treatment Protocol

**Figure 1:**
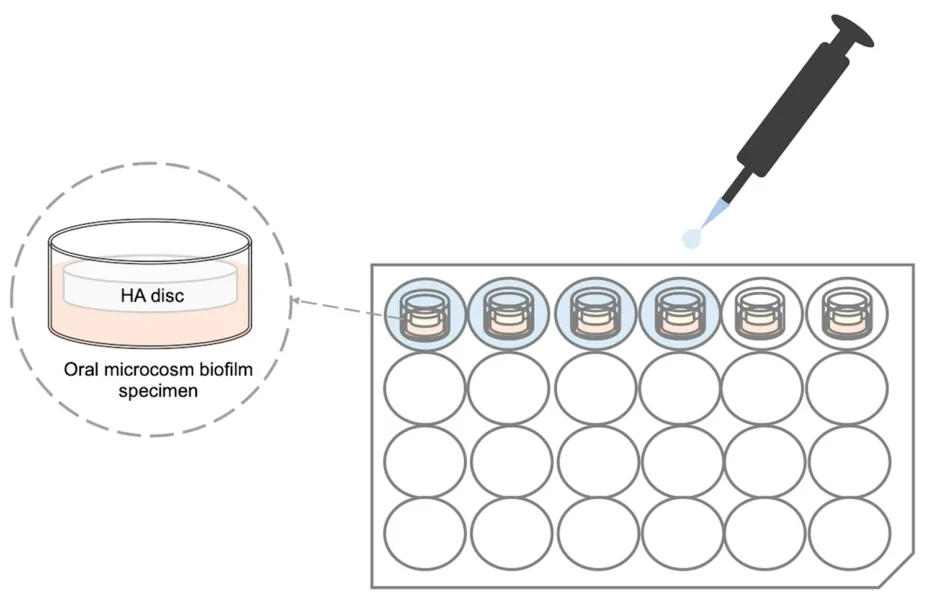
Human saliva inoculation on specimens for the formation of oral microcosm biofilms. The specimens were HA discs fixed in acrylic molds. HA, hydroxyapatite.

Hydroxyapatite (HA) discs (7 mm diameter and 1.8 mm thickness; Himed, Old Bethpage, NY, USA) were embedded in cylindrical acrylic molds and secured within dental putty (AFFINIS Putty Soft; Coltene, Altstätten, Switzerland). Each HA disc was placed in a well of a 24-well plate and immersed in 1.5 mL of fresh saliva for initial inoculation (Figure 1). The samples were incubated at 37 °C in 10% CO_2_ for 4 h. Saliva was then replaced with basal medium mucin (1.4 mL) supplemented with 0.5% sucrose (0.1 mL), and the medium was renewed daily for six consecutive days to allow biofilm maturation [26].

On day 7, mature biofilms were treated once daily for 1 min with one of the following solutions: 0.5% BCSO (experimental), 0.5% DMSO (negative control), and 0.12% CHX (positive control; Hexamedine, Bukwang Pharm Co., Ltd., Seoul, Republic of Korea). After each treatment, the samples were gently rinsed with PBS and the culture medium was replenished. This regimen was continued for 6 days (days 7–12).

### 2.6. Assessment of Red-to-Green Fluorescence Intensity of Oral Microcosm Biofilms

Biofilm images were acquired daily using a QLF-D camera (Inspektor Research Systems BV, Amsterdam, The Netherlands). All images were captured under standardized ambient conditions with the camera positioned 10 cm from the samples. For blue-light excitation, the settings were as follows: shutter speed, 1/60 s; aperture, 7.1; and ISO, 1600. The settings for the white-light images were as follows: shutter speed, 1/60 s; aperture, 8.0; and ISO 250. The images were first processed using C3 software v1.16 (Inspektor Research Systems BV) and then quantified using Image-Pro v10.0.8 (Media Cybernetics, Silver Spring, MD, USA). Red and green fluorescence intensities were measured within a predefined region of interest. Red fluorescence specifically detects endogenous porphyrins (e.g., protoporphyrin IX), which predominantly accumulate in Gram-negative, black-pigmented obligate anaerobes during the late stages of biofilm development [34,35]. Consequently, the degree of anaerobic, dysbiotic biofilm maturation, which serves as a robust proxy for pathogenic potential, was expressed as the red-to-green fluorescence intensity ratio (${\mathrm{Ratio}}_{R/G}$) [36].

### 2.7. Assessment of Aciduric Bacterial Viability

Following the treatment phase, the biofilm-formed HA disks were washed thrice with 1.5 mL of PBS to eliminate planktonic cells. The specimens were then placed in 1 mL of PBS and subjected to 1 min of vortexing (VM-96A; Lab Companion, Seoul, Republic of Korea) and 1 min of sonication (SHB-1025; Saehan Sonic, Seoul, Republic of Korea) to detach and homogenize the biofilms. The generated biofilm suspensions were serially diluted (10^−1^–10^−6^) and inoculated (100 µL) onto 5% brain heart infusion agar plates (Becton Dickinson & Co., Franklin Lakes, NJ, USA) previously acidified to a pH of 4.8. After incubation at 37 °C with 10% CO_2_ for 72 h, the aciduric bacterial colony-forming units (CFUs) were counted to determine the mean Log_10_CFUs/mL.

### 2.8. Quantification of EPS and Bacterial Biovolumes Using Confocal Laser Scanning Microscopy

To visualize EPS within the biofilms, the growth medium was supplemented with Alexa Fluor 647–dextran conjugate (Ex/Em 647/668 nm; Thermo Fisher Scientific, Scoresby, Australia), and the labeled medium was replaced daily. After 6 days of treatment, the biofilms were rinsed with PBS and removed from the HA discs by sequential vortexing and ultrasonication. Bacterial biovolumes were stained with SYTO™ 9 green-fluorescent nucleic acid stain (1 μM; Ex/Em 480/500 nm; Thermo Fisher Scientific) for 15 min in the dark. The biofilm specimens with fluorescence labeling were mounted on glass slides and examined using a confocal laser scanning microscope (CLSM; LSM 900, Carl Zeiss Microscopy, Jena, Germany) equipped with a 10× objective lens and imaging software (ZEISS ZEN Microscopy Software 3.6; Carl Zeiss). To eliminate observational bias, a quantitative analysis was performed by an independent investigator blinded to the group allocation. Using Image-Pro v10.0.8 (Media Cybernetics, Silver Spring, MD, USA), standardized fluorescence intensity thresholds and predefined regions of interest were uniformly applied across all images. In the processed images, fluorescence in the red channel represented EPS, whereas that in the green channel corresponded to bacterial biovolumes, enabling a highly reproducible estimation of the total biofilm biomass [26].

### 2.9. Statistical Analysis

Data were analyzed using SPSS Statistics v28.0 (IBM Corp., Armonk, NY, USA). Although the biofilms (*n* = 19 per group) were derived from a single saliva donor and inherently represent technical replicates, the individual, physically isolated well was strictly defined a priori as the independent experimental unit for all statistical analyses and sample size calculations [37]. This was justified by the closed-ecosystem design, wherein independent treatment application and absence of physical or chemical spill-over between wells were ensured, thereby satisfying the independence assumption required for the one-way analysis of variance (ANOVA) and Kruskal–Wallis models [38]. Prior to statistical analyses, normality was assessed using the Kolmogorov–Smirnov test, and homogeneity of variance was evaluated using Levene’s test. The MTT assay outcomes and baseline ${\mathrm{Ratio}}_{R/G}$ did not satisfy the assumption of normality (*p* < 0.05 for the Kolmogorov–Smirnov test) and were therefore analyzed using the non-parametric Kruskal–Wallis test. Post hoc pairwise comparisons were subsequently performed using the Mann–Whitney *U* test with a Bonferroni-adjusted significance level of α = 0.0083 (0.05/6). The EPS, bacterial biovolumes, aciduric bacterial counts, and post-treatment ${\mathrm{Ratio}}_{R/G}$ satisfied the assumptions of both normality (*p* > 0.05) and homogeneity of variance (*p* > 0.05 for Levene’s test). Accordingly, these variables were analyzed using ANOVA, followed by Tukey’s post hoc test. The overall level of statistical significance was set at α = 0.05. Descriptive statistics are presented as medians (25th–75th percentiles) for non-parametric data and as means ± standard deviations for normally distributed data.

## 3. Results

### 3.1. Optimal Experimental Concentration of BCSO

**Figure 2:**
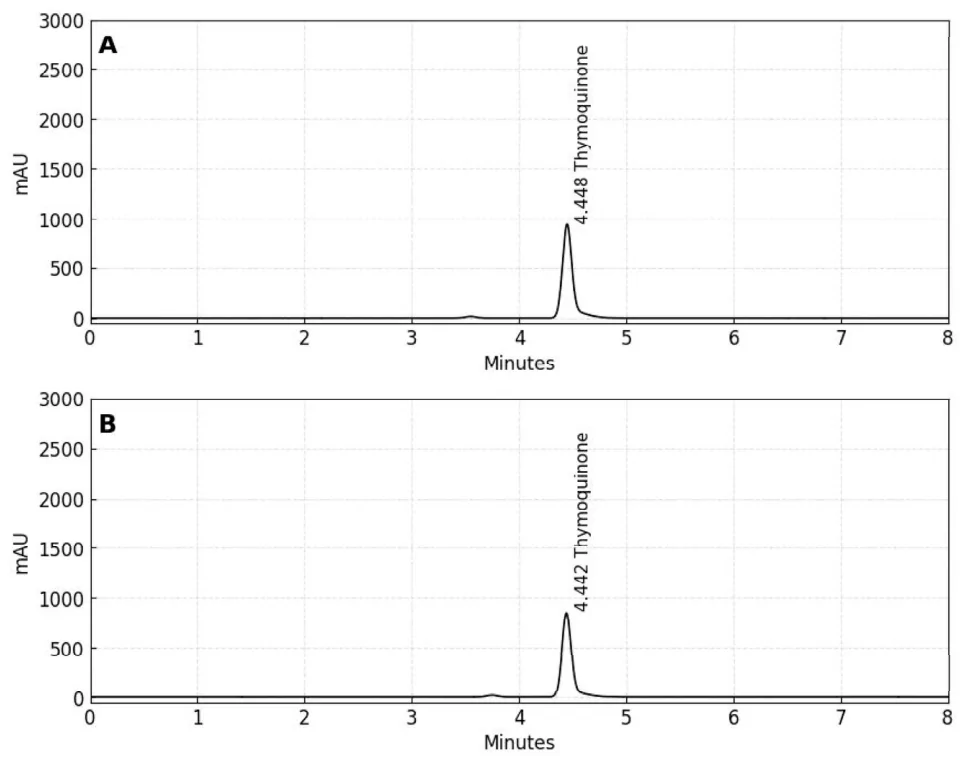
Representative HPLC chromatograms of the authentic thymoquinone reference standard (**A**) and the tested black cumin seed oil (BCSO) sample (**B**). Retention times were 4.448 min for the thymoquinone standard and 4.442 min for the BCSO sample. Chromatograms were recorded at 254 nm. HPLC, High-performance liquid chromatography.

The chromatogram of the authentic thymoquinone reference standard exhibited a distinct, well-resolved peak at a retention time of 4.448 min (Figure 2). The chromatographic profile of the BCSO sample revealed a matching predominant peak at a nearly identical retention time of 4.442 min (Figure 2). The absolute concentration of thymoquinone in BCSO was determined to be 9600 ± 130 µg/mL, based on the linear regression equation obtained from the external standard calibration curve.

**Figure 3:**
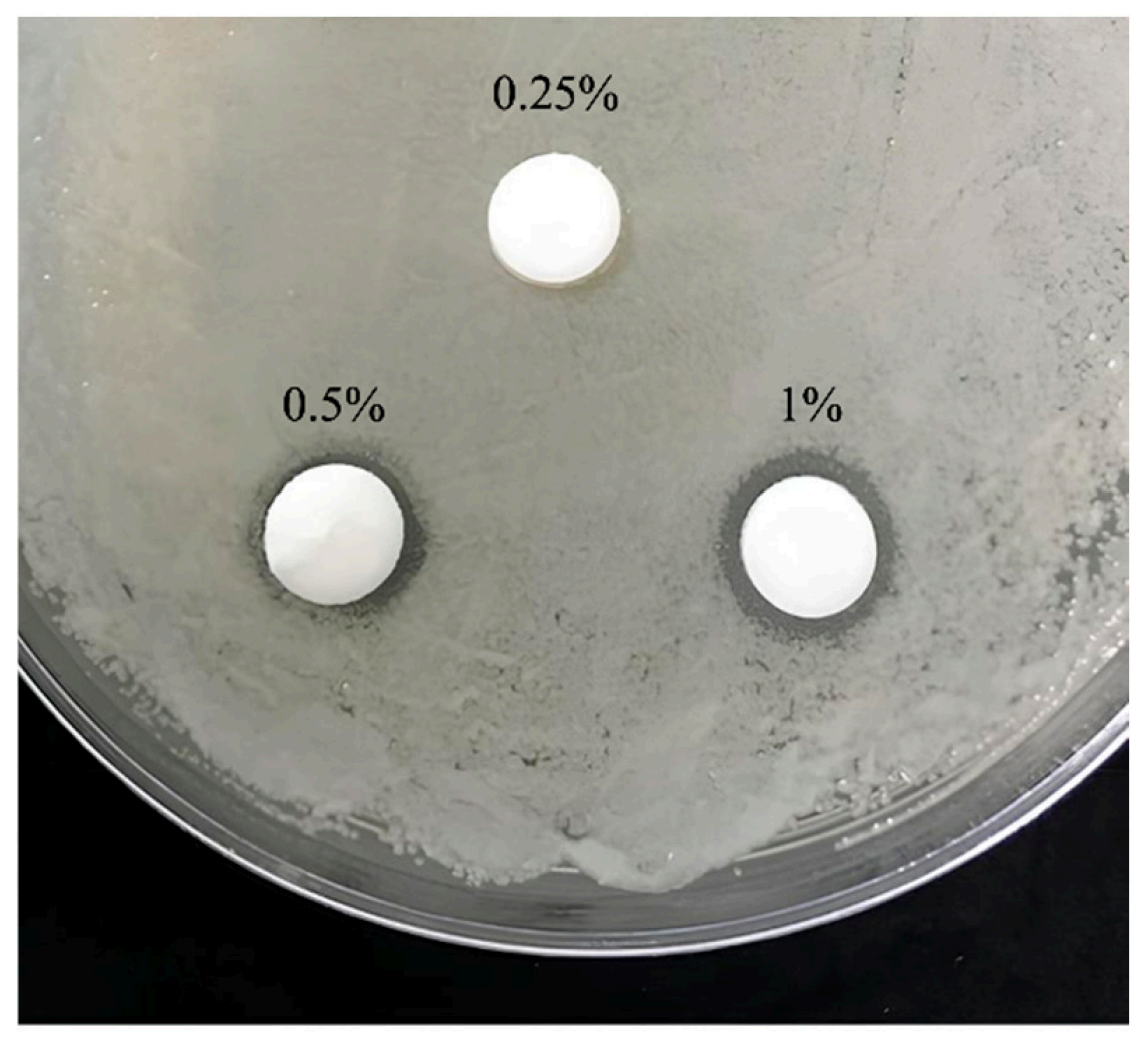
Inhibition zones of black cumin seed oil (BCSO) at varying concentrations in a disc diffusion assay. No visible inhibition zone is observed around the 0.25% BCSO disc, whereas measurable inhibition zones were observed at the 0.5% and 1% BCSO discs. BCSO, black cumin seed oil; BHI, Brain Heart Infusion.

In the disc diffusion assay, no inhibition zone was observed with 0.25% BCSO (6.00 ± 0.00 mm), whereas measurable inhibition zones were evident with both 0.5% and 1% BCSO (7.29 ± 0.29 mm and 8.25 ± 0.26 mm, respectively; Figure 3). Therefore, 0.25% BCSO was excluded as a candidate treatment concentration.

The MTT assay revealed a statistically significant difference in cell viability among the three BCSO concentrations (*p* < 0.001; Table 1). The vehicle control group (0.5% DMSO) exhibited the highest cell viability, significantly exceeding all BCSO-treated groups. While the median viability for all tested concentrations remained above the 70% threshold stipulated by the ISO standard, the 1% BCSO concentration exhibited a lower range limit (67.57%) falling below this non-cytotoxic criterion. Furthermore, subsequent post hoc analysis revealed that cell viability was significantly reduced at 1% compared with 0.5% BCSO (*p* = 0.002), whereas no significant difference was observed between 0.25% and 0.5% BCSO (*p* = 0.654). Therefore, 0.5% BCSO was selected as the optimal treatment concentration for subsequent experiments.

### 3.2. Effects of BCSO on Biofilm Biomass: EPS and Bacterial Biovolumes

**Figure 4:**
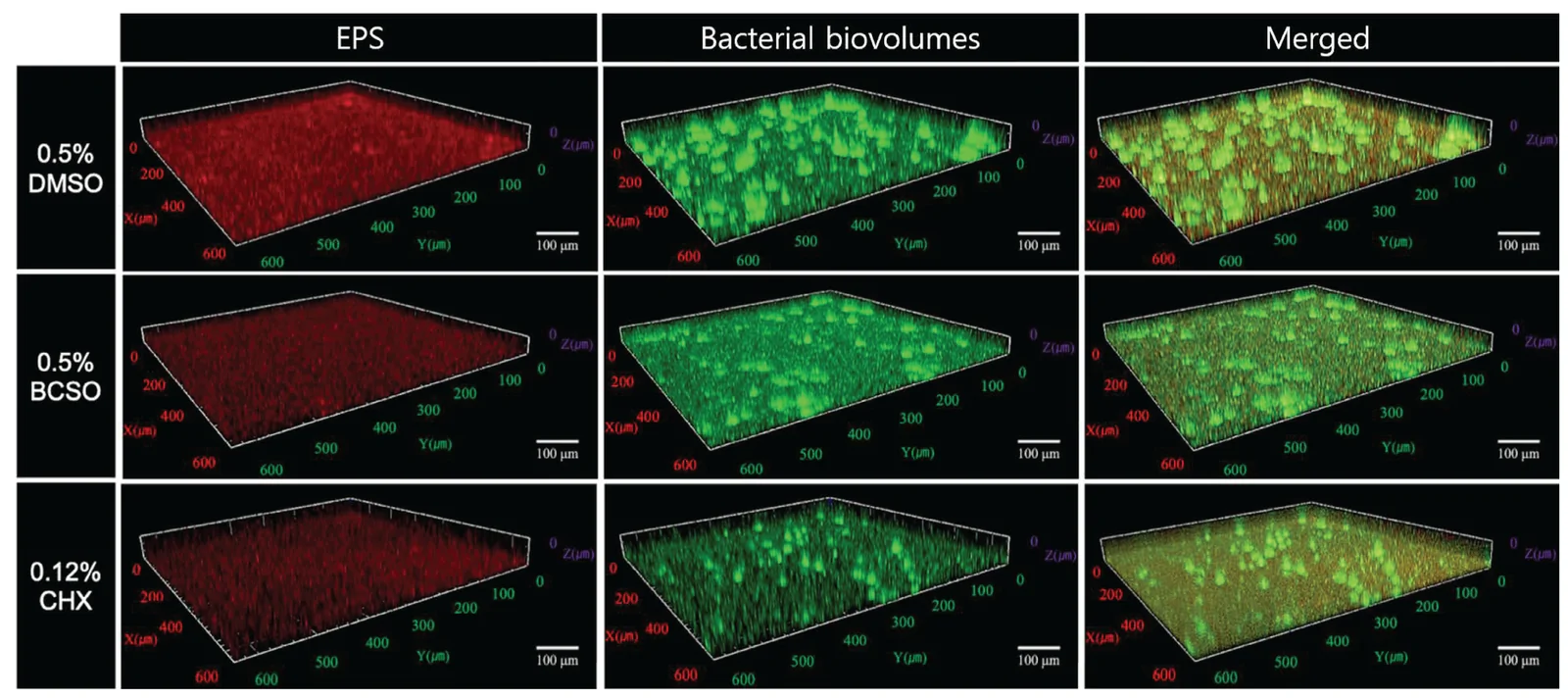
Confocal laser scanning microscopy images illustrating EPS and bacterial biovolumes within the oral microcosm biofilm. Each image represents a specific treatment group and was observed at 10× magnification. The merged images provided a combined view of EPS and bacterial biovolumes under each treatment condition. BCSO, black cumin seed oil; CHX, chlorhexidine gluconate; DMSO, dimethyl sulfoxide; EPS, extracellular polymeric substances.

The EPS biovolume differed significantly depending on the treatment agent (*p* < 0.001; Table 2). Post hoc analysis revealed that the EPS biovolume was significantly lower in the BCSO group than in the DMSO group (*p* < 0.001). In contrast, no significant difference in EPS biovolume was detected between the BCSO and CHX groups (*p* = 0.856). Similarly, the bacterial biovolume showed a significant overall difference among the treatment groups (*p* = 0.011; Table 2). Post hoc analysis demonstrated a significant reduction in bacterial biovolume for the BCSO group compared with the DMSO group (*p* = 0.045), whereas no significant difference was observed between the BCSO and CHX groups (*p* = 0.816). As shown in Figure 4, the DMSO group exhibited relatively stronger red EPS and green bacterial fluorescence signals than the other treatment groups.

### 3.3. Effects of BCSO on Aciduric Bacterial Viability

The aciduric bacterial viability differed significantly depending on the treatment agent (*p* = 0.018, Table 3). Post hoc analysis revealed a significant reduction in bacterial load for both the BCSO and CHX groups compared with the DMSO negative control (*p* = 0.028 and *p* = 0.049, respectively). In contrast, no significant difference was detected between the BCSO and CHX groups (*p* = 0.969).

### 3.4. Effects of BCSO on Red Fluorescence of Oral Microcosm Biofilms

**Figure 5:**
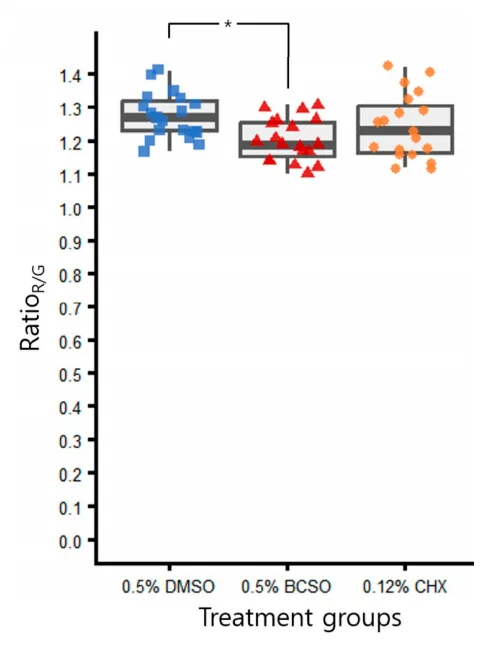
Red-to-green fluorescence intensity ratio of oral microcosm biofilms after treatment. The box plots display the median and interquartile ranges (25th–75th percentiles), with the whiskers indicating the minimum and maximum values. Individual data points are overlaid to show the data distribution. Differences between groups were evaluated using one-way analysis of variance, followed by Tukey’s post hoc test at a significance level of α = 0.05. * *p* = 0.015. BCSO, black cumin seed oil; CHX, chlorhexidine gluconate; DMSO, dimethyl sulfoxide; ${\mathrm{Ratio}}_{R/G}$, red to green fluorescence intensity ratio.

**Figure 6:**
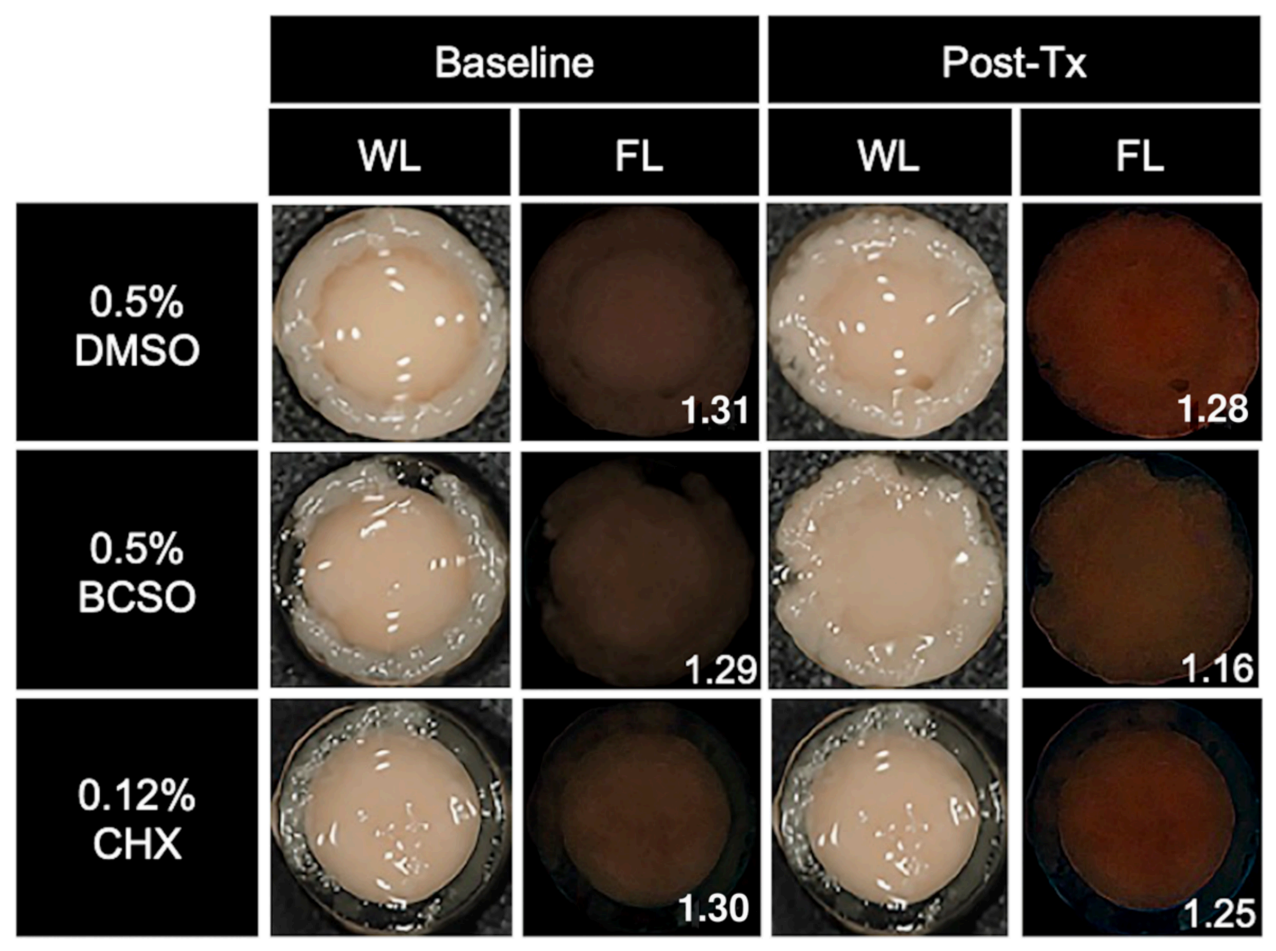
White light (WL) and fluorescence light (FL) images of oral microcosm biofilms after treatment. Each image represents a different treatment group and was captured using a quantitative light-induced fluorescence-digital camera. Baseline images correspond to 6-day-old untreated biofilms, and post-treatment (Post-Tx) images show biofilms after the final treatment. The ${\mathrm{Ratio}}_{R/G}$ values for the oral biofilms are displayed at the bottom-right corner. BCSO, black cumin seed oil; CHX, chlorhexidine gluconate; DMSO, dimethyl sulfoxide; ${\mathrm{Ratio}}_{R/G}$, red to green fluorescence intensity ratio (captured by a quantitative light-induced fluorescence-digital camera); Tx, treatment.

At baseline, no significant difference in the ${\mathrm{Ratio}}_{R/G}$ was observed among the three groups (*p* = 0.653; Table 4). Following treatment, the ${\mathrm{Ratio}}_{R/G}$ was significantly lower in the BCSO group than in the DMSO group (*p* = 0.015; Table 4; Figure 5). However, the CHX group did not show a statistically significant difference from the DMSO group (*p* = 0.399; Table 4). Furthermore, no significant difference was observed between the BCSO and CHX groups (*p* = 0.264; Table 4). In fluorescence images, biofilms treated with BCSO or CHX showed weaker red fluorescence intensity than those treated with DMSO (Figure 6).

## 4. Discussion

In this study, a 0.5% BCSO concentration was strategically selected to achieve a crucial balance between antibiofilm efficacy and host cell tolerance. Quantitative HPLC analysis established that the absolute thymoquinone content in the standardized BCSO was approximately 9600 µg/mL (Figure 2). Consequently, the 0.5% BCSO formulation delivered an effective thymoquinone dose of approximately 48 µg/mL. At this specific concentration, BCSO demonstrated substantial antipathogenic activity without inducing severe cellular stress, as evidenced by measurable inhibition zones and preservation of eukaryotic fibroblast viability. Although the 0.25% formulation yielded no measurable inhibition zone (Figure 3), increasing the concentration to 1% significantly compromised cytocompatibility compared with the 0.5% treatment (*p* = 0.002; Table 1). Viability at 0.5% exceeded the ISO-compliant non-cytotoxicity threshold of 70% [33]. Therefore, a 0.5% concentration, delivering a safe yet active thymoquinone concentration, was selected as the optimal experimental concentration, maximizing the mitigation of pathogenic microbiota without compromising host cytocompatibility.

In terms of antibiofilm efficacy, quantitative CLSM analysis revealed that both 0.5% BCSO and 0.12% CHX effectively disrupted the architecture of mature 6-day oral microcosm biofilms, resulting in a significant reduction in EPS and total bacterial biovolumes relative to the negative control (Table 2; Figure 4). To further evaluate the antibacterial efficacy against the residual biofilm community, the viability of aciduric bacteria was evaluated. Aciduric species, including *Streptococcus* and *Lactobacillus* spp., are early and middle colonizers that contribute to the massive structural scaffold of cariogenic biofilms [1,8]. Both treatments significantly reduced the aciduric bacterial load compared with the negative control (*p* = 0.018; Table 3), demonstrating a significant reduction in these matrix-producing populations.

Both agents achieved comparable reductions in structural biomass and aciduric bacterial viability. To further evaluate the suppression of biofilm maturation, the ${\mathrm{Ratio}}_{R/G}$ was assessed (Table 4; Figure 5 and Figure 6). Red fluorescence detected by QLF technology originates from endogenous porphyrins primarily synthesized by obligate anaerobes, such as *Porphyromonas* and *Prevotella* spp. [34,35]. Consequently, the ${\mathrm{Ratio}}_{R/G}$ serves as a highly specific proxy for anaerobic biofilm maturation [35]. In this regard, 0.5% BCSO significantly reduced this maturation metric compared with the negative control. Conversely, the reduction in the CHX group was not statistically significant relative to the vehicle control, suggesting that porphyrin-producing anaerobic clusters may have remained metabolically active within the CHX-treated biofilms [6,12,13].

We hypothesize that the observed outcomes regarding anaerobic maturation are heavily influenced by the distinct physicochemical properties of the agents, which govern their respective penetration capabilities into the biofilm matrix. As biofilms mature, they develop a dense EPS matrix rich in polyanionic constituents, including extracellular DNA and acidic polysaccharides, which can function as a profound ion-exchange barrier [2,24]. CHX is a dicationic molecule that preferentially binds to these negatively charged structures on the superficial layers, precipitating a strong reaction-diffusion limitation [6,39]. Consequently, cationic CHX molecules are likely sequestered and rapidly depleted through electrostatic binding with the outer aciduric populations and matrix components, thereby failing to diffuse into the deeper anaerobic niches. In contrast, thymoquinone, the principal active compound in BCSO, is an uncharged, highly lipophilic molecule [19,22]. This lipophilicity may allow thymoquinone to circumvent charge-based surface sequestration and permeate deep into the dense EPS to reach metabolically active pathogenic clusters [27,40,41].

Beyond the physical penetration capabilities governed by its lipophilicity, the sustained reduction in EPS biovolume and suppressed biofilm maturation observed in the BCSO-treated group can be further elucidated through the specific biochemical mechanisms of thymoquinone. The structural integrity of mature oral biofilms heavily relies on the continuous synthesis of EPS, primarily water-insoluble glucans catalyzed by glucosyltransferases (Gtfs) secreted by early colonizers like *S. mutans* [1,3,42]. Previous studies have demonstrated that *N. sativa* derivatives, including thymoquinone, exert multi-target antimicrobial activities that disrupt bacterial metabolic pathways [19,22,43]. Crucially, these bioactives have been shown to significantly interfere with the enzymatic activity and expression of Gtfs, thereby hindering the conversion of available sucrose into the dense, sticky glucan matrix [18,21]. This suppression of EPS synthesis deprives the microbial consortium of its essential 3D structural scaffold, leading to the destabilization of the biofilm architecture and increased susceptibility of previously protected cells [27,44]. Furthermore, thymoquinone has been reported to interfere with intercellular quorum-sensing pathways that are vital for coordinating biofilm maturation and virulence expression among diverse oral taxa [28]. By downregulating these autoinducer mechanisms, BCSO disrupts the synchronized transition of the microbial community into a highly organized, porphyrin-producing anaerobic consortium, which provides a plausible explanation for the significant reduction in the ${\mathrm{Ratio}}_{R/G}$ metric observed in our study [35].

To contextualize our phenotypic findings, recent in silico molecular docking studies offer plausible mechanistic insights [21,43]. Computational models reveal that *N. sativa* derivatives, including thymoquinone, exhibit high binding affinities to critical bacterial targets, such as Gtfs and Sortase A, which are essential for EPS biosynthesis and bacterial adherence [25,43,45]. Furthermore, they strongly interact with MurA, a fundamental enzyme for cell wall construction in oral pathogens like *S. mutans* [21,43]. While the literature suggests that thymoquinone may effectively downregulate structural matrix production by competitively binding to these active sites [21,43], we emphasize that these mechanisms remain hypothetical in the context of the present study and were not directly investigated experimentally. Rather than representing observed experimental outcomes, integrating this molecular docking literature provides a comprehensive mechanism of action supporting our phenotypic results, suggesting that BCSO may dismantle mature oral biofilms through a multi-target blockade of bacterial integrity and EPS biosynthesis pathways.

From a clinical perspective, the effective management of biofilm-associated oral diseases necessitates therapeutic strategies that guarantee sustained virulence attenuation, rather than superficial biomass removal. Long-term reliance on conventional broad-spectrum antimicrobials, such as CHX, has raised significant concerns regarding the disruption of oral microbiome homeostasis and induction of antimicrobial resistance [12,13,14]. Therefore, the potential of BCSO to comprehensively degrade dense EPS architectures and neutralize oral pathogens without causing ecological collateral damage aligns directly with the modern paradigm of preventive management. Crucially, the substantial physical disruption of the EPS deprives surviving or newly introduced early colonizers of the essential structural scaffold required for rapid recolonization [1,27]. These findings underscore the translational potential of BCSO as a pragmatic, naturally derived adjunctive therapy that can bypass the inherent diffusion limitations of charge-dependent antimicrobials.

This study had some limitations that should be considered. First, although utilizing a standardized single-donor oral microcosm biofilm model successfully minimized baseline variability to isolate specific phenotypic responses to BCSO [46,47], it inherently restricts the external validity required to capture the full inter-individual diversity of the human oral microbiome [48]. Even if ecological pressures primarily drive biofilm pathogenicity [49], this strict homogenization limits direct clinical extrapolation. Therefore, subsequent in vitro studies employing pooled saliva or multiple independent clinical donors are necessary to verify these findings across more ecologically diverse oral microenvironments. Second, regarding the ${\mathrm{Ratio}}_{R/G}$, the positive control (CHX) did not statistically separate from the negative control (DMSO). While this non-separation reflects the inherent penetration limitations of CHX in mature biofilms, it also poses a limitation of the assay and the in vitro model itself, warranting caution in the interpretability of this outcome. Third, although the putative differential penetration of BCSO and CHX aligns with their physicochemical properties, this mechanism remains a hypothesis due to the absence of direct spatial and binding analyses (e.g., z-stack intensity profiling, zeta-potential measurements). Fourth, using 0.5% DMSO as a vehicle for BCSO introduces a physicochemical disparity compared with the aqueous formulation of CHX [50]. While the aqueous CHX formulation was deliberately retained in this study to accurately reflect its gold-standard clinical application, we acknowledge that this solvent disparity prevents a perfectly controlled formulation comparison. Even at sub-inhibitory concentrations, this solvent disparity could independently modulate matrix interactions and diffusion kinetics through the polyanionic EPS. Future investigations utilizing alternative formulation strategies, such as high-intensity ultrasonication-based nanoemulsions, are required to circumvent these potential solvent-induced confounding effects. Finally, the chemical characterization of BCSO in this study was largely limited to the evaluation of thymoquinone content. Given that the botanical oil is a complex phytochemical mixture, attributing the overall antibiofilm effect exclusively to a single marker is an oversimplification, as it does not fully account for the potential synergistic interactions of other uncharacterized secondary metabolites [22,23].

Nevertheless, given the observed biomass-reducing, antibacterial, and antibiofilm effects, our results suggest that BCSO represents a promising natural adjunctive candidate that could ultimately help mitigate the long-term reliance on conventional chemical antiseptics, such as CHX, for the management of chronic oral biofilms. To facilitate the clinical translation of this approach for complex oral biofilm-associated diseases, future studies must prioritize formulation optimization, rigorous long-term safety evaluations, and direct mechanistic validation in physiologically relevant in vitro and in vivo models.

## 5. Conclusions

Within this in vitro oral microcosm model, 0.5% BCSO and 0.12% CHX effectively reduced structural biomass and the viability of aciduric bacteria. Furthermore, 0.5% BCSO significantly attenuated late-stage, porphyrin-associated anaerobic biofilm maturation relative to the negative control. Collectively, these findings provide a fundamental in vitro basis for the targeted antibiofilm properties of this natural extract. However, to avoid overextending these strictly in vitro observations, further extensive investigations are warranted. Future research must comprehensively profile global microbial viability, elucidate precise molecular mechanisms, and rigorously evaluate in vivo safety before carefully considering any clinical or translational applications.

## Figures and Tables

**Table 1:** L929 cell viability following black cumin seed oil (BCSO) treatment at varying concentrations.

Test Materials	*N*	Cell Viability (%)
0.5% DMSO	7	93.16 (90.84–95.31) ^a^
0.25% BCSO	7	81.53 (74.24–82.53) ^b^
0.5% BCSO	7	78.37 (74.37–81.53) ^b^
1% BCSO	7	72.14 (67.57–73.52) ^c^
*p*-value		<0.001

Values are expressed as medians (25th–75th percentile). The *p*-value represents the overall group comparison using the Kruskal–Wallis test. ^a,b,c^ Different superscript letters within the same column indicate statistically significant differences among the treatment groups, as determined by the Mann–Whitney *U* test with α = 0.0083.

**Table 2:** Effects of different treatments on biomass in oral microcosm biofilms.

Treatment	*N*	EPS (μm^3^/μm^2^)	Bacterial Biovolumes (μm^3^/μm^2^)
0.5% DMSO	19	18.65 ± 3.41 ^a^	29.73 ± 9.18 ^a^
0.5% BCSO	19	10.85 ± 5.11 ^b^	18.82 ± 11.22 ^b^
0.12% CHX	19	9.86 ± 3.74 ^b^	16.19 ± 8.33 ^b^
*p*-value		<0.001	0.011

Values are presented as the mean ± standard deviation. Statistical significance was assessed using one-way analysis of variance. ^a,b^ Different superscript letters within the same column indicate statistically significant differences among the treatment groups, as determined by Tukey’s post hoc test (α = 0.05). BCSO, black cumin seed oil; CHX, chlorhexidine gluconate; DMSO, dimethyl sulfoxide; EPS, extracellular polymeric substances.

**Table 3:** Mean colony-forming units of aciduric bacteria after antibacterial treatment.

Treatment	*N*	Log_10_CFUs/mL
0.5% DMSO	19	5.95 ± 0.54 ^a^
0.5% BCSO	19	5.47 ± 0.49 ^b^
0.12% CHX	19	5.51 ± 0.60 ^b^
*p*-value		0.018

Data are presented as mean ± standard deviation. The *p*-value is obtained using one-way analysis of variance. ^a,b^ Different superscript letters within the same column indicate statistically significant differences among the treatment groups, as determined by Tukey’s post hoc test at α = 0.05. BCSO, black cumin seed oil; CFUs, colony-forming units; CHX, chlorhexidine gluconate; DMSO, dimethyl sulfoxide.

**Table 4:** Red-to-green fluorescence intensity ratio (${\mathrm{Ratio}}_{R/G}$) of oral microcosm biofilm at baseline and following treatment.

Treatment	*N*	${\mathrm{Ratio}}_{R/G}$	
		Baseline	Post-Treatment
0.5% DMSO	19	1.31 (1.23–1.33)	1.27 ± 0.07 ^a^
0.5% BCSO	19	1.30 (1.23–1.31)	1.20 ± 0.06 ^b^
0.12% CHX	19	1.29 (1.20–1.32)	1.24 ± 0.10 ^ab^
*p*-value		0.653 ^†^	0.021 ^‡^

Values are expressed as the median (25th–75th percentile) or mean ± standard deviation. The *p*-value represents the overall group comparison by the Kruskal–Wallis test ^†^ or one-way analysis of variance (ANOVA) ^‡^. ^a,b^ Different superscript letters within the same column indicate statistically significant differences among the treatment groups, as determined by Tukey’s post hoc test (α = 0.05) following a significant one-way ANOVA. BCSO, black cumin seed oil; CHX, chlorhexidine gluconate; DMSO, dimethyl sulfoxide.

## Data Availability

The datasets analyzed in this study are available upon request from the corresponding author. However, owing to intellectual property rights restrictions, these data are not publicly accessible.
